# Longitudinal Association Between Oral Status and Cognitive Decline Using Fixed-effects Analysis

**DOI:** 10.2188/jea.JE20200476

**Published:** 2022-07-05

**Authors:** Sakura Kiuchi, Taro Kusama, Kemmyo Sugiyama, Takafumi Yamamoto, Upul Cooray, Tatsuo Yamamoto, Katsunori Kondo, Ken Osaka, Jun Aida

**Affiliations:** 1 Department of International and Community Oral Health, Tohoku University, Graduate School of Dentistry, Sendai, Japan; 2 Department of Dental Sociology, Kanagawa Dental University, Kanagawa, Japan; 3 Department of Social Preventive Medical Sciences, Center for Preventive Medical Sciences, Chiba University, Chiba, Japan; 4 Department of Gerontological Evaluation, Center for Gerontology and Social Science, National Center for Geriatrics and Gerontology, Aichi, Japan; 5 Department of Oral Health Promotion, Graduate School of Medical and Dental Sciences, Tokyo Medical and Dental University, Tokyo, Japan

**Keywords:** oral health, number of teeth, oral function, cognitive decline, dementia

## Abstract

**Background:**

Although the feasibility of randomized trials for investigating the long-term association between oral health and cognitive decline is low, deriving causal inferences from observational data is challenging. We aimed to investigate the association between poor oral status and subjective cognitive complaints (SCC) using fixed-effects model to eliminate the confounding effect of unobserved time-invariant factors.

**Methods:**

We used data from Japan Gerontological Evaluation Study (JAGES) which was conducted in 2010, 2013, and 2016. β regression coefficients and 95% confidence intervals [CIs] were calculated using fixed-effects models to determine the effect of deteriorating oral status on developing SCC. Onset of SCC was evaluated using the Kihon Checklist-Cognitive function score. Four oral status variables were used: awareness of swallowing difficulty, decline in masticatory function, dry mouth, and number of teeth.

**Results:**

We included 13,594 participants (55.8% women) without SCC at baseline. The mean age was 72.4 (standard deviation [SD], 5.1) years for men and 72.4 (SD, 4.9) years for women. Within the 6-year follow-up, 26.6% of men and 24.9% of women developed SCC. The probability of developing SCC was significantly higher when participants acquired swallowing difficulty (β = 0.088; 95% CI, 0.065–0.111 for men and β = 0.077; 95% CI, 0.057–0.097 for women), decline in masticatory function (β = 0.039; 95% CI, 0.021–0.057 for men and β = 0.030; 95% CI, 0.013–0.046 for women), dry mouth (β = 0.026; 95% CI, 0.005–0.048 for men and β = 0.064; 95% CI, 0.045–0.083 for women), and tooth loss (β = 0.043; 95% CI, 0.001–0.085 for men and β = 0.058; 95% CI, 0.015–0.102 for women).

**Conclusion:**

The findings suggest that good oral health needs to be maintained to prevent the development of SCC, which increases the risk for future dementia.

## INTRODUCTION

Approximately 50 million individuals are currently living with dementia worldwide.^1^ The number of cases is estimated to rise to 152 million by 2050. A total of 28.8 million disability-adjusted life years have been attributed to dementia.^2^ Mild cognitive impairment (MCI) has been identified as an important precursor of dementia.^3^ MCI is defined as the intermediate state of cognitive function between the changes seen in aging and those fulfilling the criteria for dementia and often Alzheimer’s disease.^1^^,^^3^ The prevention of MCI could potentially reduce the incidence of dementia in the future.

Recently, several studies have reported an association between poor oral status and cognitive decline or the onset of dementia.^4^^–^^8^ There are several possible biomedical and social mechanisms underlying the association between oral status and cognitive decline. Vascular dementia, which is caused by cerebrovascular diseases, such as stroke, is the second most prevalent type of dementia.^9^ Periodontal inflammation has been associated with an increased risk of cerebrovascular diseases.^10^ Furthermore, diabetes is a well-known risk factor for dementia^9^; studies have suggested that inflammation caused by periodontal disease increases the risk of diabetes.^11^ Depression is also a risk factor for dementia, and poor oral health is associated with the onset of depression.^12^ Reduced physical activity and social isolation are also well-known risk factors associated with the development dementia.^9^ These factors are also possibly associated with the oral status because poor oral conditions can impair social interactions and increase homeboundness.^13^

Despite the abundance of possible connections between oral status and cognitive decline, determining the causal relationships between them with epidemiological studies is a formidable task. Both oral disease and dementia are chronic diseases with long-term progression, and experimental studies, such as randomized control trials, are difficult for investigating the relationship. Therefore, any causal inferences have to be drawn from observational data.^14^ However, deducing causal inferences from observational data is challenging because of the bias introduced by unmeasured confounders. Recently, econometric methods are being used increasingly in healthcare research to facilitate the acquisition of robust causal inferences.^15^ Fixed-effects models have the ability to diminish the bias caused by even unmeasured time-invariant confounders.^14^^,^^16^ It is possible that previous observational studies may have overestimated the association between oral health and cognitive decline because of unmeasured confounding bias. Therefore, we examined the hypothesis that poor oral status could increase the risk of cognitive decline using the fixed-effects model.

## METHODS

### Data collection

**Figure 1.**
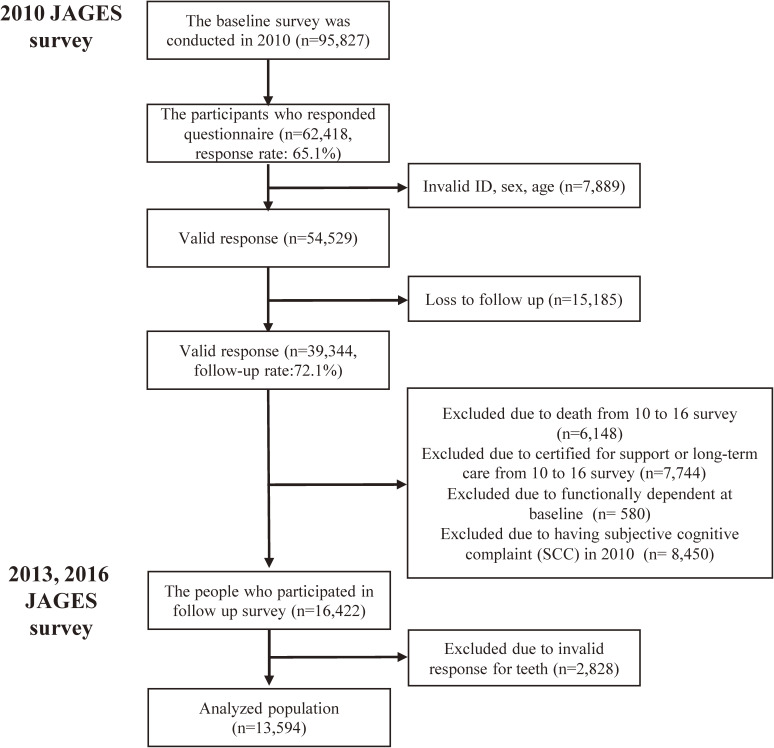
The flowchart of the participants for the analysis

This longitudinal study used data from the Japan Gerontological Evaluation Study (JAGES), an ongoing cohort study for community-dwelling older individuals aged 65 years and above and living in Japan.^17^ We used data from three waves of the survey; the data from 2010 were considered as baseline and data from 2013 and 2016 were considered as follow-up. A postal survey was randomly distributed to 95,827 individuals in 16 Japanese municipalities from August 2010 to January 2012. A total of 62,418 participants responded to the initial (baseline) survey (response rate: 65.1%). Among them, 54,529 participants were linked to 2013 and 2016 survey. However, 15,185 participants were lost to follow-up over the 6-year study period (follow-up rate: 72.1%). A total of 6,148 deaths were observed, and 7,744 participants became ineligible for the survey because they moved away from the community dwellings. We focused on participants who were functionally independent and did not have subjective cognitive complaints (SCC) at baseline. Therefore, we excluded 580 functionally dependent participants and 8,450 participants with SCC. After excluding participants with invalid responses for the teeth variable, 13,594 participants were included in this analysis. The flowchart is shown in Figure 1.

### The outcome variable

We used SCC as the outcome measure in this study. SCC was determined from three questions related to cognitive function in the Kihon Checklist (KCL-CF).^18^ Tomata et al (2017) reported higher KCF-CF score was related to a higher risk of the onset of dementia.^19^ Following questions were included in the KCL-CF.

1. “Do your family or your friends point out your memory loss?”
2. “Do you make a call by looking up phone numbers?”
3. “Do you find yourself not knowing today’s date?”

Participants were asked to answer ‘yes’ or ‘no’ to the above-mentioned questions. Answer ‘yes’ for the 1^st^ and 3^rd^ questions, and answer ‘no’ for the 2^nd^ question was indicative of cognitive decline. Participants with at least one response indicating cognitive decline were considered to have SCC.^20^

### Explanatory variables

We used swallowing difficulty, decline in masticatory function, dry mouth, and number of teeth as explanatory variables. Swallowing difficulty, decline in masticatory function, and dry mouth were assessed using responses to the following questions, respectively.

1. “Have you often choked on your tea or soup recently?”
2. “Do you have any difficulties eating tough foods compared to 6 months ago?”
3. “Do you often experience having a dry mouth?”

These questions were also a part of the KCL-CF questionnaire.^18^

The number of teeth were measured by asking participants, “How many remaining teeth do you have?”. Participants were asked to choose between four categories (≥20, 10–19, 1–9, or 0) in 2010 and 5 categories (≥20, 10–19, 5–9, 1–4, or 0) in 2013 and 2016. In this study, two category classification for number of teeth (ie ≥20 or 0–19) was used. This categorization was obtained from a previous study.^21^ We conducted sensitivity analysis with different number of teeth categories (*N* = 14,830).

### Covariates

Potential confounders were selected based on a systematic review.^5^ Demographic factors including age (65–69, 70–74, 75–79, 80–84, or ≥85 years) and marital status (married, living together/single, divorced, or widowed) were recorded. Socioeconomic status was evaluated using the equivalent household income (<1.00, 1.00–1.99, 2.00–2.99, 3.00–3.99, or ≥4.00 million Japanese Yen) and education level (≤9, 10–12, or ≥13 years of education). A previous study that used fixed-effects analysis treated educational level as a time-invariant variable^16^; however, we treated it as time-variant because lifelong learning has become normal in Japan.^22^ The equivalent household income was calculated by dividing the annual household income by the square root of the number of family members. Comorbidities included the presence of hypertension (yes or no) and diabetes mellitus (yes or no). Health behaviors included alcohol consumption (current drinker, past drinker, or nondrinker), smoking history (current smoker, past smoker, or non-smoker), and daily walking time (<30 minutes, 30–59 minutes, 60–89 minutes, or ≥90 minutes). We did not adjust for the history of stroke, depression, social relationship, and the denture use. We considered these variables to be mediators between oral status and cognitive decline because it was reported that poor oral status increased the risk of stroke,^23^ depression,^24^ and less communication,^25^ and denture use.^26^

### Statistical analysis

First, the suitability of the fixed-effects models over the random-effects models was confirmed using the Hausman test.^27^ A linear probability model with the onset of SCC as the outcome was used to improve interpretability.^28^^,^^29^ We calculated the β coefficient with a 95% confidence interval (CI) to investigate how changes in oral status affected the probability of onset of SCC. The following equation was used to calculate the β coefficients.

$$\begin{array}{rl} & \text{Subjective cognitive complaint }it\\ & \;=\beta 0+\beta 1\text{ swallowing difficulty }it\\ & \;+\beta 2\text{ masticatory function }it\\ & \;+\beta 3\text{ dry mouth }it\\ & \;+\beta 4\text{ number of teeth }it\\ & \;+\beta k\;Xk\;it+Ai+Uit \end{array}$$

The *subjective cognitive complaint* (0,1) of individual “*i*” at a given time “*t*” is the left part of this formula. Four time-variant explanatory variables for the *i*^th^ individual at time *t* are included on the right side. *Xk* is the matrix of time-variant confounding variables. *Ai* models the fixed-effect, ie, the effect of unmeasured time-invariant variables such as personal ability. *Uit* is an error term. The β coefficient indicated the degree of the probability of the onset of SCC when oral status increase by one unit, in other words, from 0 (no) to 1 (yes) for oral functions or 0 (≥20 teeth) to 1 (0–19 teeth) for number of teeth.

We found that 3,732 participants responded with a higher value for the number of teeth than that of the previous wave. We dropped these variables and performed multivariate imputations (MI). We also conducted a sensitivity analysis that excluded the data of participants with 0–19 teeth could not change their dental status over 6 years, which might not have been suitable for the fixed-effects analysis. Therefore, we excluded the data of the participants with 0–19 teeth at baseline for the sensitivity analysis (*N* = 6,666).

Cognitive decline was found to be affected by sex.^30^ Therefore, we conducted an analysis stratified by sex. The oral status variables were assessed using separate models because they were highly correlated, especially between severe tooth loss and chewing difficulty.^31^ Swallowing difficulty, decline in masticatory function, dry mouth, and number of teeth were separately included separately with the age variable in model 1. Marital status, equivalent household income, education level, presence of hypertension, presence of diabetes mellitus, drinking history, smoking history, and walking time were added in model 2.

We compensated the missing values with the iterative imputation method (missForest) based on a random forest using Python.^32^ We conducted MI in a wide data format in line with the recommendations of Young et al (2015).^33^ We used Stata 15.0 MP (Windows version; Stata Corp, College Station, TX, USA) for all analyses except MI. The reporting of this study conforms to STROBE guidelines.

### Ethical approval

The Ethics Committee of the National Center for Geriatrics and Gerontology (approval number: 992), Chiba University (approval number: 2493) and Tohoku University Graduate School of Medicine (21-40) provided ethical approval for the JAGES. Participants were notified that participation was voluntary and returning a completed survey was considered as consent for enrollment.

## RESULTS

Table 1 presents the baseline characteristics of 13,594 participants (6,006 men and 7,588 women) after MI. The distribution showed similar before and after MI (the baseline characteristics before MI are shown in eTable 1). The mean age of the participants at baseline was 72.4 (SD, 5.1) years for men and 72.4 (SD, 4.9) years for women. The age of the study population ranged from 65 to 96 years at baseline. Table 2 shows the cross-tabulation of the onset of SCC in 2016 by oral status in 2010 and changes in 2010–2016. Within the follow-up period, 26.6% of men and 24.9% of women developed SCC. Participants who reported comparatively lower levels of oral status at baseline and no changes in oral status from 2010 to 2016 tended to have a higher prevalence of SCC in 2016.

**Table 1.** Descriptive baseline characteristics of the participants who did not have any subjective cognitive complaint (2010) (*N* = 13,594)

		Men (*N* = 6,006)	Women (*N* = 7,588)
	
**Explanatory variable**		%	%
Swallowing difficulty	No	89.0	89.4
	Yes	11.0	10.6
Decline in masticatory function	No	80.1	80.5
	Yes	19.9	19.5
Dry mouth	No	85.3	86.4
	Yes	14.7	13.6
Number of teeth	≥20	49.1	49.0
	0–19	50.9	51.0
**Covariates**			
Age, years	65–69	35.1	33.9
	70–74	33.5	35.6
	75–79	20.9	21.3
	80–84	8.4	7.5
	≥85	2.0	1.7
Marital status	Single, Divorced, Widowed	11.0	35.2
	Married, Living together	89.0	64.8
Income, million Japanese yen	<1.00	6.8	14.3
	1.00–1.99	33.8	34.1
	2.00–2.99	29.0	27.5
	3.00–3.99	17.9	13.6
	≥4.00	12.5	10.4
Educational level, years	≤9	37.0	46.5
	10–12	35.4	38.7
	≥13	27.5	14.7
Hypertension	No	56.8	54.4
	Yes	43.2	45.6
Diabetes mellitus	No	85.9	90.8
	Yes	14.1	9.2
Drinking history	Current drinker	61.8	16.7
	Past drinker	4.2	0.8
	Non-drinker	34.0	82.5
Smoking history	Current smoker	17.8	2.7
	Past smoker	55.4	4.2
	Non-smoker	26.8	93.0
Walking time	<30 minutes	24.2	28.6
	30–59 minutes	37.2	37.7
	60–89 minutes	19.0	15.9
	≥90 minutes	19.6	17.7
Total		100.0	100.0

Note: The descriptive characteristics shown here used imputed datasets.

**Table 2.** The cross-tabulation of the onset of subjective cognitive complaint in 2016 by oral health status in 2010 and changes in 2010–2016 (*N* = 13,594)

		Men			Women		
		Total	No	Yes	Total	No	Yes
		*N*	%	%	*N*	%	%
**Oral status in 2010**							
Swallowing difficulty	No	5,345	74.4	25.6	6,786	75.9	24.1
	Yes	661	64.8	35.2	802	68.5	31.5
Decline in masticatory function	No	4,813	75.4	24.6	6,105	76.7	23.3
	Yes	1,193	65.1	34.9	1,483	68.7	31.3
Dry mouth	No	5,124	75.1	24.9	6,555	76.2	23.8
	Yes	882	63.3	36.7	1,033	68.5	31.5
Number of teeth	≥20	2,949	75.8	24.2	3,717	77.1	22.9
	0–19	3,057	71.0	29.0	3,871	73.2	26.8
**Changes in oral status in 2010–2016**							
Swallowing difficulty	Improve	287	67.6	32.4	346	73.4	26.6
	No change	5,092	75.5	24.5	6,407	76.6	23.4
	Decline	627	58.4	41.6	835	64.9	35.1
Decline in masticatory function	Improve	448	71.4	28.6	522	71.6	28.4
	No change	4,555	74.7	25.3	5,874	76.6	23.4
	Decline	1,003	68.3	31.7	1,192	69.6	30.4
Dry mouth	Improve	432	66.7	33.3	409	71.6	28.4
	No change	4,945	74.9	25.1	6,270	76.6	23.4
	Decline	629	66.1	33.9	909	66.3	33.7
Number of teeth	No change	5,613	73.4	26.6	7,243	75.2	24.8
	Decline	393	73.0	27.0	345	73.0	27.0
Total		6,006	73.4	26.6	7,588	75.1	24.9

Table 3 presents the sex-stratified results of the fixed-effects models generated to predict the onset of SCC. The probability of the onset of SCC was significantly higher when participants acquired swallowing difficulty (β = 0.088; 95% CI, 0.065–0.111 for men and β = 0.077; 95% CI, 0.057–0.097 for women), decline in masticatory function (β = 0.039; 95% CI, 0.021–0.057 for men and β = 0.030; 95% CI, 0.013–0.046 for women), dry mouth (β = 0.026; 95% CI, 0.005–0.048 for men and β = 0.064; 95% CI, 0.045–0.083 for women) and tooth loss (β = 0.043; 95% CI, 0.001–0.085 for men and β = 0.058; 95% CI, 0.015–0.102 for women). This result can be interpreted as follows. Men who acquired swallowing difficulty demonstrated 8.8% (95% CI, 6.5–11.1%) higher probability of developing SCC on average. The probability was the highest among participants who acquired swallowing difficulty compared to those with a decline in masticatory function, dry mouth, and tooth loss. All other covariate specific coefficients and 95% CI are presented in eTable 2 and eTable 3.

**Table 3.** Probability of the onset of subjective cognitive complaints (95% confidence interval) by oral status using fixed-effects linear regression analysis from the data of 2010, 2013, 2016 panels (*N* = 13,594)

	Model 1		Model 2	
**Men (*N* = 6,006)**	β	95% CI	β	95% CI
Swallowing difficulty	0.089	(0.066–0.112)^***^	0.088	(0.065–0.111)^***^
Decline in masticatory function	0.037	(0.019–0.055)^***^	0.039	(0.021–0.057)^***^
Dry mouth	0.025	(0.003–0.046)^*^	0.026	(0.005–0.048)^*^
Tooth loss^a^	0.042	(0.000–0.084)^*^	0.043	(0.001–0.085)^*^
**Women (*N* = 7,588)**				
Swallowing difficulty	0.077	(0.058–0.097)^***^	0.077	(0.057–0.097)^***^
Decline in masticatory function	0.030	(0.014–0.046)^***^	0.030	(0.013–0.046)^***^
Dry mouth	0.065	(0.047–0.084)^***^	0.064	(0.045–0.083)^***^
Tooth loss^a^	0.062	(0.019–0.106)^**^	0.058	(0.015–0.102)^**^

CI, confidence interval.

Model 1: Swallowing difficulty, decline in masticatory function, dry mouth and tooth loss were separately included in the models with age.

Model 2: Model 1 + marital status, income, education level, hypertension, diabetes mellitus, drinking history, smoking history, and walking time.

^a^Reference category: ≥20.

^*^P < 0.05.

^**^P < 0.01.

^***^P < 0.001.

In the results of both of the sensitivity analysis with a different number of teeth categories and without participants with 0–19 teeth at baseline, the β coefficient of tooth loss was slightly larger than the main analysis (shown in eTable 4 and eTable 5).

## DISCUSSION

To the best of our knowledge, this was the first study to examine the effect of a wider range of oral status variables on SCC using large-panel data, after adjusting for unmeasured time-invariant confounders. The probabilities of the onset of SCC were higher when participants acquired swallowing difficulty (differences in the onset were 8.8% for men and 7.7% for women), decline in masticatory function (3.9% for men and 3.0% for women), dry mouth (2.6% for men and 6.4% for women), and tooth loss (4.3% for men and 5.8% for women) on average. These results are important for understanding the risk of dementia because the SCC was related to the increased risk of incident dementia.^19^

The longitudinal association observed between tooth loss and future dementia in this study is consistent with the findings of previous studies.^5^^,^^6^ We added robust evidence obtained from fixed-effects model, which nullified the confounding effect of unmeasured time-invariant variables. Our findings related to masticatory function were consistent with those of previous systematic reviews.^4^^,^^7^ Earlier studies have suggested that patients with dementia tended to have dysphagia^34^ and dry mouth^35^; however, no study has examined the longitudinal association between swallowing difficulty or dry mouth and the onset of SCC among the community-dwelling older adults.

Several plausible mechanisms are underlying the association between poor oral status and cognitive decline. First, the deterioration in oral status affects the quality of social activities such as speaking and smiling. Tooth loss and deterioration of oral status can predict future homeboundness among the older population.^13^ Physical inactivity and social isolation are major risks for dementia.^9^ Second, a deteriorating oral condition could increase the risk of depression due to the lack of social activities,^13^^,^^36^ subsequently increasing the risk of dementia.^9^ Third, the decline of oral status could lead to malnutrition, which is a possible cause of cognitive decline among older adults.^37^ Those with dry mouth tend to avoid dry food, such as bread and vegetables.^38^ Swallowing difficulty causes malnutrition because they reduce or alter food intake from solids to liquids.^39^ A previous study has shown that vitamin deficiency partially explains the association between malnutrition and cognitive decline.^40^ Furthermore, an association between nutritional biomarkers at baseline and cognitive decline at follow-up has also been reported.^41^ Fourth, the decline in oral status might be related to cognitive decline through diminished cerebral blood flow. A systematic review that examined the results of biological experiments suggested that masticatory function may play a protective role in maintaining cognitive function.^7^ Finally, the inflammatory pathway of periodontal diseases might be related to cognitive decline.^42^ Cytokines produced by periodontal inflammation could enter the systemic circulation, which may contribute to cognitive decline.^42^

We provided evidence of the importance of maintaining good oral health as implication. Public health interventions that focus on the prevention of dental caries and periodontal diseases, which are the major causes of tooth loss,^43^ are essential. These interventions are also aid in maintaining masticatory function.^31^ The use of several medications can lead to dry mouth, which is a concern in the medical field.^44^ Therefore, reducing any unnecessary medications is required via cooperation with medical doctors.

This study has several strengths. First, we used data from a large-scale cohort from rural and urban areas of Japan. Moreover, the follow-up rate for all participants was relatively high (72.1%). Second, we used fixed-effects analysis, which enabled the reduction of the bias introduced by unobserved time-invariant confounders, such as individual personalities or abilities for keeping oral hygiene.^27^ The limitation of the study is that the fixed-effects model cannot control for unobserved time-variant variables.^27^ However, we adjusted the models using various time-variant covariates. Second, fixed-effects models cannot determine reverse causality.^27^ However, we restricted the target population to individuals without SCC at baseline. Therefore, it is unlikely that participants with cognitive impairment would have poor oral status. Third, we used a self-reported questionnaire that relied on subjective answers. Tomata et al mentioned only KCF-CF score is not sufficient for predicting dementia due to a false-negativity rate of KCF-CF.^19^ Therefore, more validated questionnaire would be expected. For explanatory variables, the self-reported number of teeth is a reliable measurement^45^ and several studies commonly used.^21^^,^^24^ We assessed the swallowing difficulty by obtaining a single question. Currently, EAT-10, the validated self-reported questions were introduced.^46^ Although a single question was used in several studies,^47^^–^^49^ future studies should consider multiple-question surveys, such as EAT-10. Additionally, we asked the participants about dry mouth using a single question. A visual analog scale measure would be a better predictor for detecting dry mouth.^50^

Fourth, our data excluded participants who were certified as having dementia by the city government or died during the follow-up. We think that the longitudinal association between oral health and the onset of SCC would have been stronger if we had included individuals with dementia or who died in our analysis.

### Conclusion

The decline in oral status (swallowing difficulty, decline in masticatory function, dry mouth, and tooth loss) was significantly associated with the onset of SCC after adjusting for time-invariant confounding factors. The present findings show the importance of keeping good oral health for preventing SCC, which is related with higher risk of dementia.

## SUPPLEMENTARY MATERIAL

eTable 1. Descriptive baseline characteristics of the participants who did not have any subjective cognitive complaints (2010) with missing values (N = 13,594)

eTable 2. Probability of the onset of subjective cognitive complaints (95% confidence interval) by oral status among men using fixed-effects linear regression analysis from the data of 2010, 2013, 2016 panels (N = 6,006)

eTable 3. Probability of the onset of subjective cognitive complaints (95% confidence interval) by oral status among women using fixed-effects linear regression analysis from the data of 2010, 2013, 2016 panels (N = 7,588)

eTable 4. Probability of the onset of subjective cognitive complaints by oral status with different number of teeth categories using fixed-effects linear regression analysis from the data of 2010, 2013, 2016 panels (N = 14,830)

eTable 5. Probability of the onset of subjective cognitive complaints (95% confidence interval) by oral status without participants who responded with 0-19 teeth at baseline using fixed-effects linear regression analysis from the data of 2010, 2013, 2016 panels (N = 6,666)
