# Supplementary material for: Longitudinal Association Between Oral Status and Cognitive Decline Using Fixed-effects Analysis
Source: J Epidemiol. 2022 Jul 5;32(7):330–6. doi: 10.2188/jea.JE20200476 (PMC9189315; doi:10.2188/jea.JE20200476)
Supplement: Supplementary file 1 [file je-32-330-s001.pdf]

eTable 1. Descriptive baseline characteristics of the participants who did not have any subjective cognitive complaints (2010) with missing values (N=13,594)

		Men (N=6,006)	Women (N=7,588)
**Explanatory variable**		%	%
Swallowing difficulty	No	88.1	88.0
	Yes	11.0	10.6
	Missing	0.9	1.4
Decline in masticatory function	No	79.4	79.3
	Yes	19.9	19.5
	Missing	0.7	1.2
Dry mouth	No	83.8	84.0
	Yes	14.7	13.6
	Missing	1.5	2.4
Number of teeth	≥20	48.8	48.2
	0–19	50.4	50.1
	Missing	0.8	1.6
**Covariates**			
Age, years	65–69	35.1	33.9
	70–74	33.5	35.6
	75–79	20.9	21.3
	80–84	8.4	7.5
	≥85	2.0	1.7
Marital status	Single, Divorced, Widowed	11.0	34.9
	Married, Living together	88.3	63.9
	Missing	0.7	1.2
Income (million Japanese yen)	<1.00	6.2	12.4
	1.00–1.99	30.8	26.9
	2.00–2.99	25.5	20.1
	3.00–3.99	17.5	13.2
	≥4.00	12.3	10.1
	Missing	7.7	17.2
Educational level, years	≤9	36.5	45.7
	10–12	35.2	38.3

	≥13	27.3	14.5
	Missing	1.0	1.5
Hypertension	No	33.7	33.8
	Yes	38.1	40.6
	Missing	28.2	25.6
Diabetes mellitus	No	58.0	65.2
	Yes	13.9	9.2
	Missing	28.2	25.6
Drinking history	Current drinker	61.5	16.5
	Past drinker	4.2	0.8
	Non–drinker	33.7	81.1
	Missing	0.6	1.6
Smoking history	Current smoker	17.5	2.7
	Past smoker	54.6	4.2
	Non–smoker	26.2	86.5
	Missing	1.7	6.7
Walking time	<30 minutes	23.7	27.2
	30–59 minutes	34.6	34.6
	60–89 minutes	19.0	15.9
	≥90 minutes	19.4	17.5
	Missing	3.3	4.8
Total		100.0	100.0

Note: Descriptive characteristics are shown here using the original datasets.

eTable 2. Probability of the onset of subjective cognitive complaints (95% confidence interval) by oral status among men using fixed-effects linear regression analysis from the data of 2010, 2013, 2016 panels (N= 6,006)

		Model 1		Model 2		Model 3		Model 4	
		β	95% CI	β	95% CI	β	95% CI	β	95% CI
Swallowing difficulty		0.088	(0.065–0.111)***						
Decline in masticatory function				0.039	(0.021–0.057)***				
Dry mouth						0.026	(0.005–0.048)*		
Tooth loss[a]								0.043	(0.001–0.085)*
Age, years[b]	70–74	0.167	(0.149–0.185)***	0.169	(0.151–0.187)***	0.169	(0.151–0.187)***	0.168	(0.150–0.186)***
	75–79	0.305	(0.281–0.329)***	0.306	(0.282–0.331)***	0.309	(0.284–0.333)***	0.306	(0.281–0.331)***
	80–84	0.461	(0.428–0.493)***	0.462	(0.430–0.495)***	0.467	(0.434–0.499)***	0.463	(0.429–0.496)***
	≥85	0.676	(0.629–0.723)***	0.677	(0.630–0.724)***	0.684	(0.637–0.731)***	0.680	(0.632–0.727)***
Married, Living together[c]		–0.019	(–0.064–0.026)	–0.021	(–0.066–0.024)	–0.022	(–0.067–0.023)	–0.020	(–0.065–0.024)
Income, million Japanese yen[d]	1.00–1.99	–0.032	(–0.061–0.004)*	–0.032	(–0.061–0.003)*	–0.033	(–0.061–0.004)*	–0.032	(–0.061–0.004)*
	2.00–2.99	–0.051	(–0.082–0.020)**	–0.052	(–0.083–0.021)**	–0.053	(–0.084–0.022)**	–0.052	(–0.084–0.021)**
	3.00–3.99	–0.064	(–0.100–0.029)***	–0.064	(–0.100–0.029)***	–0.066	(–0.101–0.030)***	–0.066	(–0.101–0.030)***
	≥4.00	–0.073	(–0.113–0.032)***	–0.072	(–0.113–0.031)**	–0.074	(–0.115–0.034)***	–0.073	(–0.114–0.032)***
Education level, years[e]	10–12	0.002	(–0.028–0.032)	0.002	(–0.028–0.032)	0.003	(–0.027–0.033)	0.003	(–0.027–0.033)
	≥13	–0.012	(–0.049–0.026)	–0.011	(–0.049–0.026)	–0.010	(–0.048–0.027)	–0.011	(–0.048–0.027)
Hypertension		–0.012	(–0.038–0.013)	–0.011	(–0.036–0.015)	–0.010	(–0.035–0.016)	–0.010	(–0.035–0.015)
Diabetes mellitus		0.027	(–0.011–0.066)	0.028	(–0.011–0.066)	0.028	(–0.011–0.066)	0.028	(–0.011–0.066)

Drinking history[f]	Past drinker	0.061	(0.031–0.090)***	0.061	(0.032–0.091)***	0.061	(0.032–0.091)***	0.062	(0.032–0.091)***
	Non–drinker	0.012	(–0.020–0.045)	0.013	(–0.020–0.045)	0.011	(–0.021–0.044)	0.012	(–0.020–0.044)
Smoking history[g]	Past smoker	0.016	(–0.022–0.054)	0.017	(–0.021–0.055)	0.019	(–0.019–0.057)	0.019	(–0.019–0.057)
	Non–smoker	0.102	(0.060–0.143)***	0.104	(0.063–0.145)***	0.104	(0.063–0.146)***	0.104	(0.063–0.145)***
Walking time[h]	30–59 minutes	–0.006	(–0.025–0.013)	–0.007	(–0.026–0.013)	–0.008	(–0.027–0.012)	–0.008	(–0.027–0.011)
	60–89 minutes	–0.011	(–0.034–0.013)	–0.013	(–0.036–0.011)	–0.013	(–0.037–0.010)	–0.014	(–0.037–0.010)
	≥90 minutes	–0.006	(–0.032–0.020)	–0.007	(–0.033–0.019)	–0.008	(–0.034–0.018)	–0.008	(–0.034–0.018)
cons		–0.091	(–0.157–0.024)**	–0.089	(–0.155–0.023)**	–0.084	(–0.151–0.018)*	–0.103	(–0.172–0.034)**

CI, confidence interval.

Model 1: Swallowing difficulty, age, marital status, income, educational level, hypertension, diabetes mellitus, drinking history, smoking history and walking time included in the models. Model 2: Model1 + decline in masticatory function instead of swallowing difficulty. Model 3: Model1 +dry mouth instead of swallowing difficulty. Model 4: Model1 + tooth loss instead of decline in swallowing difficulty.

[a] Reference category: ≥20

[b] Reference category: 65-69

[c] Reference category: Single, Divorced, Widowed

[d] Reference category: <1.00

[e] Reference category: ≤9

[f] Reference category: Current drinker

[g] Reference category: Current smoker

[h] Reference category: <30 minutes

*p< 0.05

**p< 0.01

***p<0.001

eTable 3. Probability of the onset of subjective cognitive complaints (95% confidence interval) by oral status among women using fixed-effects linear regression analysis from the data of 2010, 2013, 2016 panels (N=7,588)

		Model 1		Model 2		Model 3		Model 4	
		β	95% CI	β	95% CI	β	95% CI	β	95% CI
Swallowing difficulty		0.077	(0.057–0.097)***						
Decline in masticatory function				0.030	(0.013–0.046)***				
Dry mouth						0.064	(0.045–0.083)***		
Tooth loss[a]								0.058	(0.015–0.102)**
Age, years[b]	70–74	0.155	(0.139–0.171)***	0.157	(0.141–0.173)***	0.156	(0.140–0.172)***	0.156	(0.140–0.172)***
	75–79	0.297	(0.276–0.318)***	0.300	(0.279–0.321)***	0.298	(0.277–0.319)***	0.300	(0.279–0.321)***
	80–84	0.451	(0.424–0.479)***	0.457	(0.429–0.485)***	0.454	(0.426–0.482)***	0.456	(0.428–0.484)***
	≥85	0.626	(0.586–0.667)***	0.633	(0.592–0.674)***	0.631	(0.591–0.672)***	0.633	(0.593–0.674)***
Married, Living together[c]		-0.047	(–0.073–0.022)***	–0.048	(–0.074–0.023)***	–0.048	(–0.073–0.022)***	–0.049	(–0.074–0.023)***
Income, million Japanese yen[d]	1.00–1.99	0.005	(–0.015–0.024)	0.005	(–0.015–0.024)	0.005	(–0.014–0.025)	0.005	(–0.014–0.025)
	2.00–2.99	-0.017	(–0.038–0.005)	–0.016	(–0.038–0.006)	–0.015	(–0.037–0.007)	–0.016	(–0.037–0.006)
	3.00–3.99	-0.014	(–0.041–0.013)	–0.013	(–0.040–0.014)	–0.012	(–0.039–0.015)	–0.013	(–0.040–0.014)
	≥4.00	-0.018	(–0.049–0.014)	–0.018	(–0.049–0.013)	–0.017	(–0.048–0.015)	–0.017	(–0.049–0.014)
Education level, years[e]	10–12	-0.005	(–0.029–0.018)	–0.006	(–0.030–0.018)	–0.006	(–0.030–0.017)	–0.006	(–0.030–0.018)
	≥13	-0.017	(–0.047–0.013)	–0.018	(–0.048–0.012)	–0.018	(–0.048–0.012)	–0.018	(–0.048–0.012)
Hypertension		0.014	(–0.008–0.037)	0.015	(–0.007–0.038)	0.015	(–0.008–0.037)	0.015	(–0.008–0.038)
Diabetes mellitus		0.016	(–0.024–0.055)	0.013	(–0.026–0.053)	0.012	(–0.027–0.051)	0.013	(–0.026–0.053)

Drinking history[f]	Past drinker	0.018	(–0.024–0.060)	0.017	(–0.025–0.060)	0.016	(–0.026–0.058)	0.018	(–0.025–0.060)
	Non–drinker	0.028	(–0.002–0.058)	0.027	(–0.003–0.057)	0.026	(–0.004–0.056)	0.026	(–0.004–0.056)
Smoking history[g]	Past smoker	-0.012	(–0.095–0.071)	–0.011	(–0.094–0.072)	–0.010	(–0.092–0.073)	–0.009	(–0.092–0.074)
	Non–smoker	0.087	(–0.002–0.177)	0.089	(0.000–0.179)	0.089	(–0.001–0.179)	0.090	(0.000–0.179)
Walking time[h]	30–59 minutes	0.004	(–0.012–0.019)	0.004	(–0.012–0.019)	0.003	(–0.012–0.019)	0.004	(–0.012–0.019)
	60–89 minutes	0.007	(–0.013–0.026)	0.007	(–0.013–0.027)	0.007	(–0.013–0.027)	0.006	(–0.014–0.026)
	≥90 minutes	0.005	(–0.017–0.027)	0.006	(–0.016–0.028)	0.005	(–0.017–0.027)	0.005	(–0.017–0.027)
cons		-0.183	(–0.278–0.088)***	–0.182	(–0.277–0.087)***	–0.184	(–0.279–0.089)***	–0.206	(–0.303–0.108)***

CI, confidence interval.

Model 1: Swallowing difficulty, age, marital status, income, educational level, hypertension, diabetes mellitus, drinking history, smoking history and walking time included in the models. Model 2: Model1 + decline in masticatory function instead of swallowing difficulty. Model 3: Model1 +dry mouth instead of swallowing difficulty. Model 4: Model1 + tooth loss instead of decline in swallowing difficulty.

[a] Reference category: ≥20

[b] Reference category: 65–69

[c] Reference category: Single, Divorced, Widowed

[d] Reference category: <1.00

[e] Reference category: ≤9

[f] Reference category: Current drinker

[g] Reference category: Current smoker

[h] Reference category: <30 minutes

*p< 0.05

**p< 0.01

***p<0.001

eTable 4. Probability of the onset of subjective cognitive complaints by oral status with different number of teeth categories using fixed-effects linear regression analysis from the data of 2010, 2013, 2016 panels (N=14,830)

	Model 1		Model 2	
**Men (N=6,609)**	β	95% CI	β	95% CI
Swallowing difficulty	0.086	(0.064–0.108)***	0.085	(0.063–0.106)***
Decline in masticatory function	0.037	(0.020–0.055)***	0.040	(0.023–0.058)***
Dry mouth	0.034	(0.014–0.055)**	0.036	(0.016–0.057)**
Tooth loss[a]	0.061	(0.020–0.103)**	0.058	(0.016–0.099)**
**Women (N=8,221)**				
Swallowing difficulty	0.074	(0.055–0.093)***	0.073	(0.054–0.092)***
Decline in masticatory function	0.035	(0.019–0.051)***	0.035	(0.019–0.051)***
Dry mouth	0.062	(0.044–0.080)***	0.061	(0.043–0.079)***
Tooth loss[a]	0.062	(0.018–0.106)**	0.061	(0.017–0.105)**

CI, confidence interval.

Model 1: Swallowing difficulty, decline in masticatory function, dry mouth and tooth loss were separately included in the models with age.

Model 2: Model 1 + marital status, income, education level, hypertension, diabetes mellitus, drinking history, smoking history, and walking time.

[a] Reference category: ≥10

*p< 0.05

**p< 0.01

***p<0.001

eTable 5. Probability of the onset of subjective cognitive complaints (95% confidence interval) by oral status without participants who responded with 0-19 teeth at baseline using fixed-effects linear regression analysis from the data of 2010, 2013, 2016 panels (N=6,666)

	Model 1		Model 2	
**Men (N=2,949)**	β	95% CI	β	95% CI
Swallowing difficulty	0.093	(0.060–0.127)***	0.092	(0.058–0.126)***
Decline in masticatory function	0.042	(0.01–0.073)*	0.042	(0.010–0.073)**
Dry mouth	0.027	(–0.005–0.058)	0.028	(–0.003–0.060)
Tooth loss[a]	0.059	(0.017–0.101)**	0.060	(0.018–0.102)**
**Women (N=3,717)**				
Swallowing difficulty	0.060	(0.031–0.089)***	0.060	(0.031–0.089)***
Decline in masticatory function	0.047	(0.018–0.075)**	0.045	(0.017–0.073)**
Dry mouth	0.045	(0.018–0.072)**	0.043	(0.016–0.070)**
Tooth loss[a]	0.076	(0.033–0.119)**	0.070	(0.026–0.113)**

CI, confidence interval.

Model 1: Swallowing difficulty, decline in masticatory function, dry mouth and tooth loss were separately included in the models with age.

Model 2: Model 1 + marital status, income, education level, hypertension, diabetes mellitus, drinking history, smoking history, and walking time.

[a] Reference category: ≥20

*p< 0.05

**p< 0.01

***p<0.001
